# Neutropenic Fever–Associated Admissions Among Patients With Solid Tumors Receiving Chemotherapy During the COVID-19 Pandemic

**DOI:** 10.1001/jamanetworkopen.2023.4881

**Published:** 2023-03-27

**Authors:** Courtney J. Baus, Broc Kelley, Elizabeth Dow-Hillgartner, Christos E. Kyriakopoulos, Lucas T. Schulz, Alexander J. Lepak, Noelle K. LoConte

**Affiliations:** 1 University of Wisconsin School of Pharmacy, Madison; 2 University of Wisconsin School of Medicine and Public Health, Madison; 3 University of Wisconsin Carbone Cancer Center, Madison; 4 Department of Medicine, University of Wisconsin School of Medicine and Public Health, Madison

## Abstract

This cohort study examines the rates of neutropenic fever–associated admissions and outpatient antibiotic use among patients with cancer receiving chemotherapy before and during the COVID-19 pandemic.

## Introduction

Neutropenic fever (NF) occurs in up to 30% of patients with cancer receiving chemotherapy,^1^ affecting more than 60 000 patients annually in the US.^2^ Only 50% of NF cases have identified causes, but all patients with NF receive empirical antibiotics.^1,2^ During the COVID-19 pandemic, nonpharmaceutical interventions (NPIs) were instituted to limit the transmission of COVID-19.^3^ These NPIs also were associated with decreased rates of common seasonal respiratory viruses.^4^ COVID-19 provided a natural experiment of whether such mitigation strategies may also be associated with reduced rates of NF. In this cohort study, we aimed to determine the association of COVID-19–focused NPIs with the rates of NF-associated admissions and outpatient antibiotic use among patients with cancer who were receiving chemotherapy.

## Methods

We performed this single-center, retrospective cohort study comparing hospitalization and outpatient antibiotic use before the COVID-19 pandemic (September 2018 through February 2020) vs the COVID-19 pandemic period (March 2020 through August 2021). This study was determined to be exempt from institutional review board review by the University of Wisconsin. Consent was not obtained because the data were anonymous, per the policy of the University of Wisconsin Health Sciences institutional review board. This study conforms to the Strengthening the Reporting of Observational Studies in Epidemiology (STROBE) reporting guideline for cohort studies.^5^ Eligible participants were adults from UWHealth with solid tumor diagnoses receiving chemotherapy with a risk of NF. We did not include patients with hematologic malignant entities because their disease would be expected to increase the risk of NF beyond the risk associated with chemotherapy. All NF-associated admissions were recorded.^2^ The sample size was derived from the number of admissions during the study period. No patients admitted during the study period were excluded. Outpatient antimicrobial prescriptions, demographic data, and details of cancer diagnosis and chemotherapy were collected. All data were obtained from the electronic health record by 2 trained reviewers (C.J.B. and L.T.S.) to minimize bias. Discrete data were compared using Pearson χ^2^ tests, and nondiscrete data were compared using *t* tests. Statistical significance was set at 2-tailed *P* < .05. Analyses were done in November 2022 using Excel software version 2013 (Microsoft).

## Results

Demographics are presented in the Table. This study involved 3966 patients admitted before COVID-19 (mean age, 59.25 years [range, 19.00-90.00 years]; 50 patients hospitalized for NF; 29 men [58.00%]) and 4317 patients admitted during COVID-19 (mean age, 52.11 years [range, 28.00-76.00 years]; 27 patients hospitalized for NF; 7 men [25.93%]). The average hospital census size for the oncology service did not decrease over the study period. The patients admitted during COVID-19 were more likely to be female and were younger than those admitted before COVID-19. There was no difference in metastatic disease, number of chemotherapy cycles, or days since chemotherapy between the 2 groups. The use of granulocyte colony-stimulating factor was not significantly different. Before COVID-19, the rate of NF-associated admissions among the at-risk patients was 1.26% (50 of 3966 patients). During the COVID-19 period, this rate decreased to 0.63% (27 of 4317 patients). There was no significant difference in the rate of NF admissions early in the pandemic (March 2020 to September 2020, 18 NF cases of 504 admissions [3.6%]) compared with the second half of the first year of the pandemic (October 2020 to March 2021, 13 NF cases of 484 admissions [2.7%]). There were no deaths during hospitalization for either period, and no patients were lost to follow-up. Antimicrobial use was not significantly different across the 2 periods (Figure). Internal data (not shown) showed a decrease in positive respiratory virus testing (20.0% before the pandemic vs 7.6% during the COVID-19 pandemic), with no increase in amount of testing performed. The amount of outpatient oral antibiotics for NF treatment did not change over the study period, at less than 5% of patients in each period.

**Table.** Demographic and Clinical Characteristics of Cohort

Characteristic	Participants, No. (%)		
	Before COVID-19 (n = 3966)	During COVID-19 (n = 4317)	*P* value
Hospitalization for neutropenic fever	50 (1.26)	27 (0.63)	<.001
Age, mean (range), y^a^	59.25 (19.00-90.00)	52.11 (28.00-76.00)	.02
Sex^a^			
Male	29 (58.00)	7 (25.93)	<.001
Female	21 (42.00)	20 (74.07)	
Metastatic cancer^a^	24 (53.30)	15 (57.70)	.70
Chemotherapy cycle, mean (range), No.^a^	1.40 (1.00-6.00)	2.12 (1.00-8.00)	.06
Granulocyte colony-stimulating factor use^a^	28 (62.22)	17 (62.96)	.95
Time from chemotherapy to admission, mean (range), d^a^	9.63 (5.00-17.00)	10.42 (6.00-20.00)	.35
Palliative intent^a^	23 (51.11)	17 (62.96)	.33

^a^ Data are only for patients hospitalized with neutropenic fever, not the entire cohort.

**Figure.**
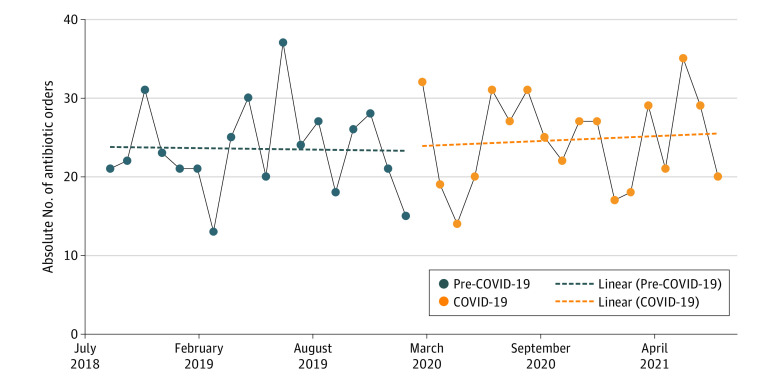
Outpatient Antimicrobial Consumption for Neutropenic Fever Graph shows the absolute number of outpatient antibiotic orders over the study period.

## Discussion

In this cohort study, among patients with solid tumors receiving chemotherapy with a risk of neutropenia, there was a significant reduction in NF-associated admission rates during the COVID-19 pandemic. Antimicrobial use and use of granulocyte colony-stimulating factor were unchanged between the study periods, so we infer from our results that the reduction in NF-associated admissions may be attributable to the use of NPIs and that NPIs may be beneficial in patients undergoing chemotherapy with a risk of neutropenia. Outpatient NF management was similar between the time periods.

The limitations of this study include that we cannot account for herd behavior changes in response to COVID-19 (eg, heightened awareness of illness symptoms, more testing and isolation, and reporting of symptoms), as well as change in counseling to patients during that same time. Future studies should analyze whether NPIs applied at the time of neutropenia for patients receiving chemotherapy are associated with decreased risk of NF.

## Notes

Supplement.
Data Sharing Statement
