# Supplementary material for: Neutropenic Fever–Associated Admissions Among Patients With Solid Tumors Receiving Chemotherapy During the COVID-19 Pandemic
Source: JAMA Netw Open. 2023 Mar 27;6(3):e234881. doi: 10.1001/jamanetworkopen.2023.4881 (PMC10043746; doi:10.1001/jamanetworkopen.2023.4881)
Supplement: Supplement. — Data Sharing Statement [file jamanetwopen-e234881-s001.pdf]

Data Sharing Statement

Baus. Neutropenic Fever-Associated Admissions Among Patients With Solid Tumors Receiving Chemotherapy During the COVID-19 Pandemic. *JAMA Netw Open*. Published March 27, 2023. doi:10.1001/jamanetworkopen.2023.4881

Data
Data available: Yes
Data types: Deidentified participant data, Data (not involving human participants)
How to access data: ns3@medicine.wisc.edu
When available: With publication

Supporting Documents
Document types: None

Additional Information
Who can access the data: Researchers requesting the data
Types of analyses: Replicating study findings
Mechanisms of data availability: with investigator support
